# Effectiveness of Neurorehabilitation in Improving the Functional Recovery and Quality of Life of Patients With Parkinson's Disease: A Case Report

**DOI:** 10.7759/cureus.52021

**Published:** 2024-01-10

**Authors:** Pinky D Israni, Vaishnavi Yadav, Anam R Sasun

**Affiliations:** 1 Cardiovascular and Respiratory Physiotherapy, Ravi Nair Physiotherapy College, Datta Meghe Institute of Higher Education and Research, Wardha, IND; 2 Neurophysiotherapy, Ravi Nair Physiotherapy College, Datta Meghe Institute of Higher Education and Research, Wardha, IND

**Keywords:** neurorehabilitation, exergames, virtual reality, physiotherapy, physical therapy, case report, outcome measures, voluntary movements, parkinson's disease

## Abstract

Parkinson's disease (PD) is a degenerative neurological illness characterized by various motor and non-motor symptoms that can lead to varying degrees of functional impairment. Key neuropathological findings include Lewy bodies containing synuclein and the loss of dopaminergic neurons in the substantia nigra, reducing the facilitation of voluntary movements. The disease is marked by bradykinesia, rigidity, and tremors. Here, we present the case of a 56-year-old man who sought neurorehabilitation due to tremors, slowness of movements, and weakness. The rehabilitation plan was carefully devised with weekly goals. The rehabilitation spanned six weeks, during which the individual showed positive improvement in all measured outcomes. Virtual reality and exergame technologies have emerged as prominent tools for enhancing balance and gait in PD. Our study utilized outcome measures such as the Unified Parkinson's Disease Rating Scale, the World Health Organization Quality-of-Life Scale, and the Barthel Index. Neurophysiotherapy plays a significant role in enhancing a patient's functional rehabilitation.

## Introduction

Parkinson's disease (PD), an age-associated degenerative neurological disorder, manifests a spectrum of motor and non-motor symptoms with variable functional implications [1]. Given the aging demographic, the anticipated rise in PD prevalence, as the second most prevalent progressive neurological ailment in the elderly, is imminent. The underlying pathology involves the degeneration of dopaminergic neurons in the midbrain's substantia nigra, accompanied by the formation of Lewy bodies, hallmark characteristics of idiopathic Parkinson's disease (IPD). Various risk factors include advanced age, exposure to pesticides, environmental pollutants, and a familial predisposition (e.g., synthetic heroin use), although the precise etiology remains elusive. Clinical presentations commonly feature bradykinesia, stiffness, rest tremors, and a stooped posture, along with a spectrum of non-motor symptoms. PD has also been associated with neurobehavioral disorders such as depression and anxiety, cognitive impairments including dementia, as well as autonomic dysregulation manifesting as hyperhidrosis and orthostasis. The intricate interplay of these multifaceted symptoms underscores the complexity of PD, necessitating a comprehensive understanding of effective management strategies [2].

Physical therapy (PT) emerges as a crucial therapeutic modality for individuals who have PD. While contemporary interventions are continually evolving, conventional physiotherapeutic approaches have undergone comprehensive exploration [3]. Over the years, physiotherapy has yielded immediate benefits for patients with PD. Deep brain stimulation has emerged as the best treatment paradigm. Successful outcomes in both translational and reverse translational neuroscience have been observed. Robust, meticulously designed, randomized controlled trials employing refined methodologies and comprehensive reporting are imperative for evaluating the enduring efficacy and economic viability of PT in PD management [4]. Despite advancements in pharmacological and surgical interventions, individuals grappling with PD confront escalating disability. Physiotherapy, grounded in movement rehabilitation, seeks to optimize functional capacity, mitigate secondary complications, and foster an educational environment supportive of holistic well-being. Improved quality of life is a result of increased freedom, safety, and general welfare, which is a positive outcome of physiotherapy rehabilitation. Empirical evidence underscores the efficacy of physiotherapy in ameliorating PD-related challenges, yet uncertainties persist regarding the most efficacious physiotherapeutic interventions [5]. In the context of PD, the significance of non-motor symptoms is progressively escalating, prompting a shift toward recognizing both motor and non-motor symptoms as integral supportive criteria. The etiology of PD remains largely elusive, with genetic risk factors acknowledged to exist. Noteworthy instances include rare monogenetic causes within non-selective populations, constituting approximately 5-10% of PD cases. The hereditary component in PD patients underscores the complexity of its origins and highlights the need for continued exploration. There are numerous environmental variables linked to an increased risk of PD [6]. Motor assessment in PD encompasses four primary domains: gait/walking, arm and hand function, balance and posture, and clinimetrics. Clinimetric evaluation plays a pivotal role, with notable scales such as the Unified Parkinson's Disease Rating Scale and the Hoehn and Yahr stages providing quantifiable insights into disease progression.

For balance and posture assessment, sophisticated techniques involving posturometric platforms are employed alongside established clinometric scales like the Berg Balance Scale (BBS), Tinetti test, and timed up and go test [7]. Investigative efforts are underway to explore potential neuroprotective factors in early-stage PD, including nicotine use, coffee consumption, and urate levels. Randomized studies are scrutinizing these factors to ascertain their impact on disease trajectory [8]. PD frequently manifests in postural instability stemming from axial motor involvement. Beyond late-onset IPD and atypical Parkinsonian syndromes, postural dysfunction often heralds clinical deterioration in advanced disease stages [9]. Bradykinesia, characterized by "slowness of movement," stands as a prominent and widely recognized symptom of PD. Clinical manifestations of bradykinesia encompass weakness, tremors, and rigidity, contributing to the complex clinical picture of this neurodegenerative disorder [10].

## Case presentation

Patient information

A 56-year-old male presented at the neurorehabilitation center with complaints of generalized weakness, slowness of movement, and involuntary upper limb movements. He also reported difficulty in walking, impacting his daily activities. Three months prior, the patient experienced falls in his washroom, and the involuntary movements in his right upper limb gradually intensified, particularly during periods of heightened anxiety. The patient has a 10-year history of hypertension and noted an increased frequency of micturition. 

Clinical findings

**Figure 1:**
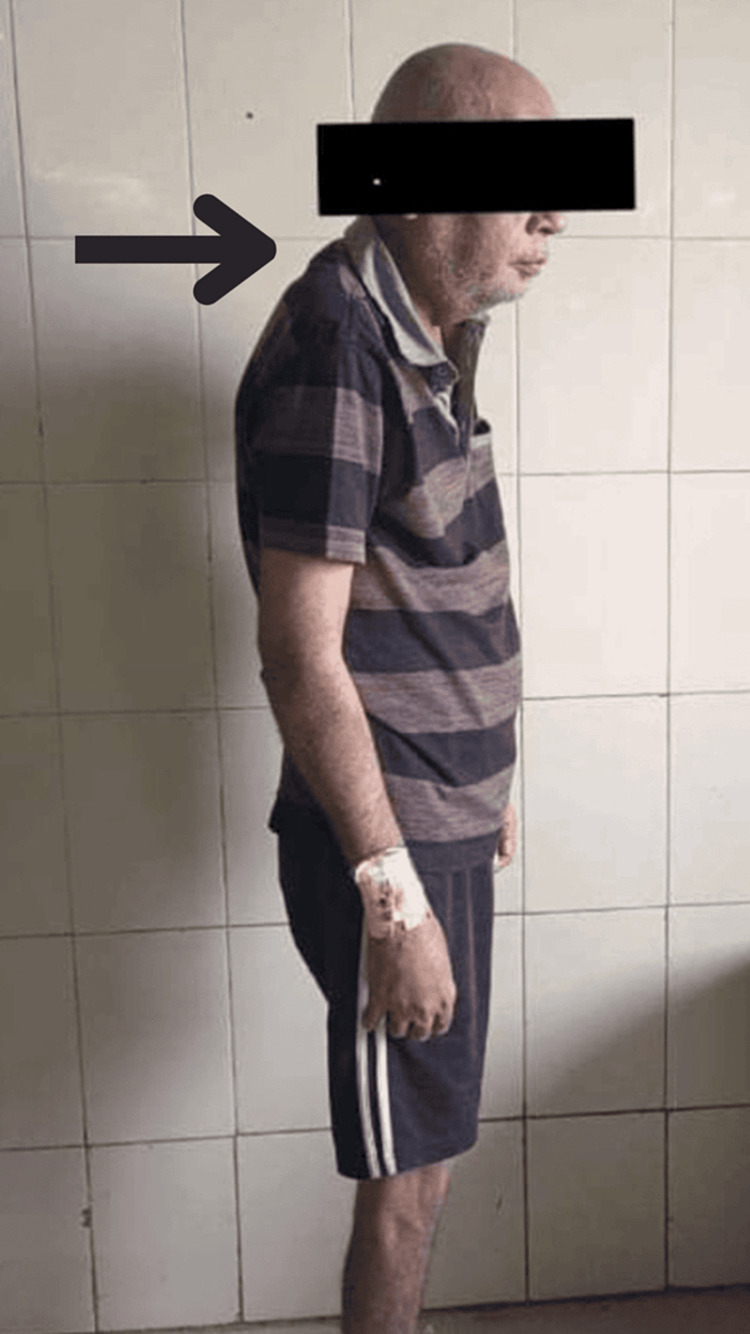
Posture examination in lateral view The black arrow shows the kyphotic-forward head

Following the patient's oral consent, a comprehensive assessment was undertaken. Notable observations included involuntary tremors in the right arm and a gradual progression of slowness of movement. The patient also presented with a slightly masked face. Posture assessment revealed a kyphotic-forward head posture, illustrated in Figure 1. On reflex examination, the knee jerk was exaggerated on the right side. Additionally, the tone assessment highlighted increased tone in wrist flexors, ulnar deviators, and finger flexors.

Therapeutic intervention

**Figure 2:**
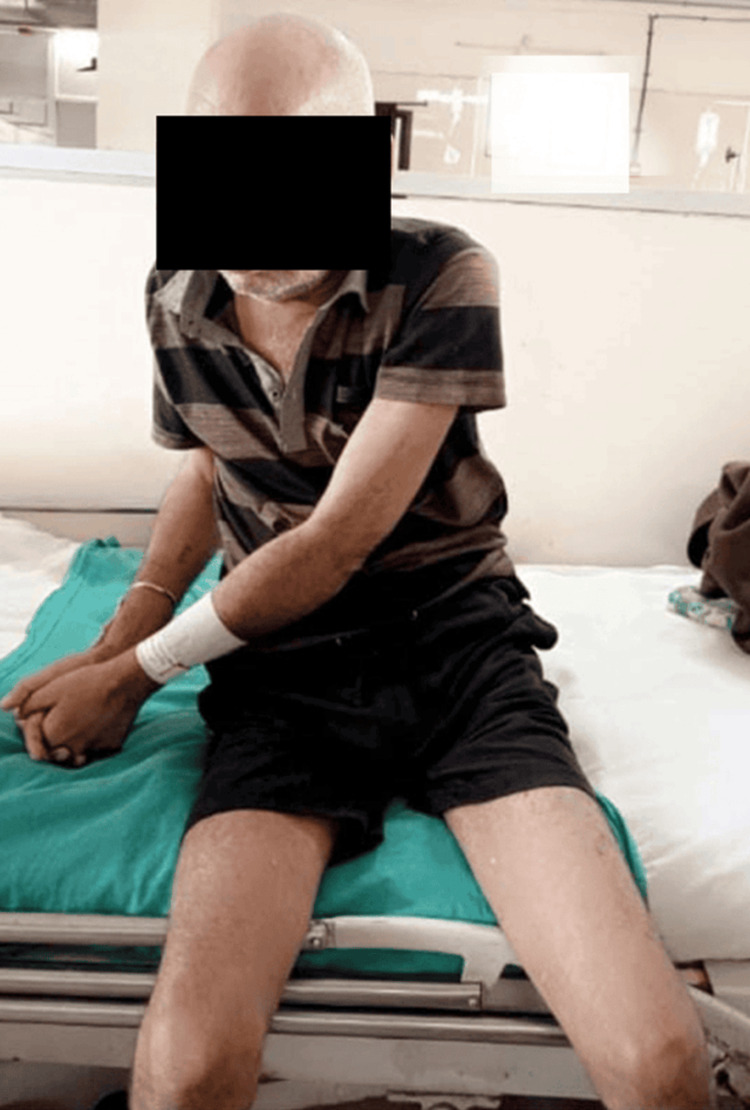
PNF D2 flexion of the upper limb PNF: proprioceptive neuromuscular facilitation

**Figure 3:**
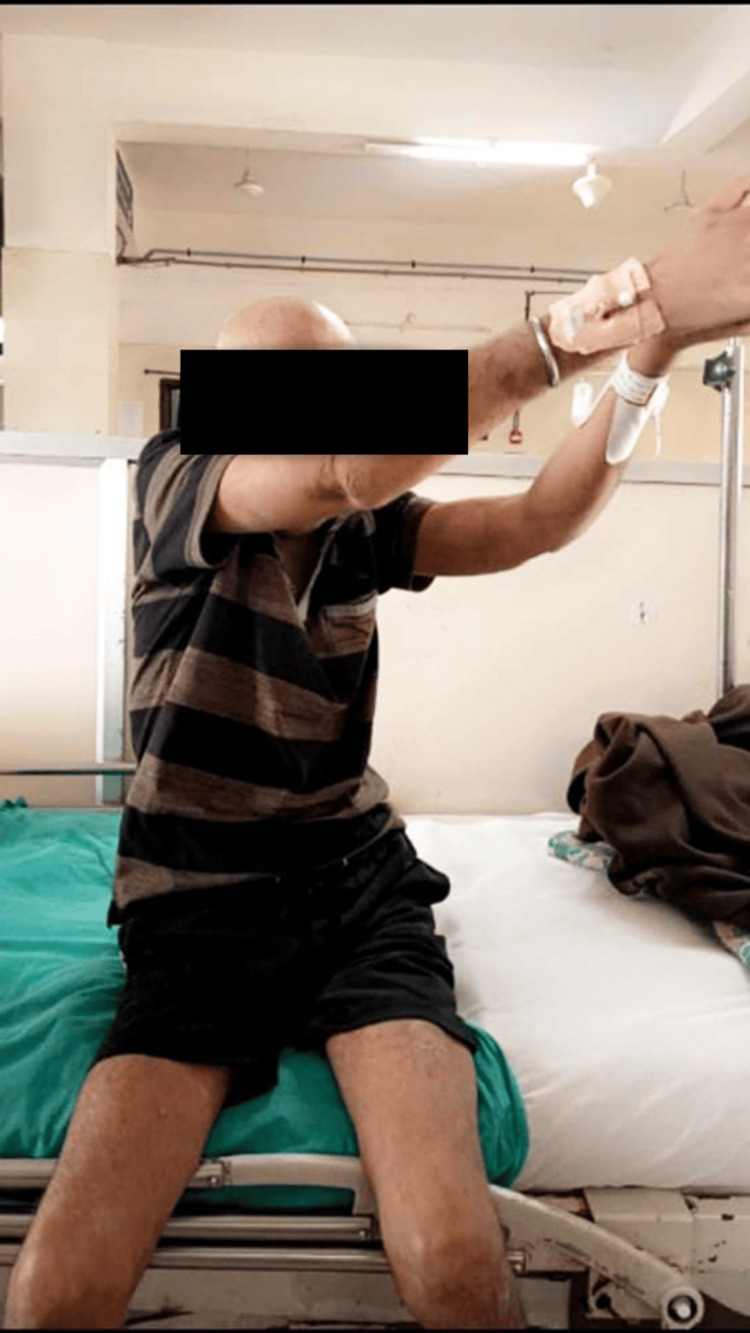
Performing D2 extension of the upper limb PNF: proprioceptive neuromuscular facilitation

Table 1 summarizes the therapeutic intervention given to the patient. Figure 2 and Figure 3 show the patient performing proprioceptive neuromuscular facilitation of the upper limb.

**Table 1:** Therapeutic intervention PNF: proprioceptive neuromuscular facilitation; mins: minutes; reps: repetitions

Goals	Interventions
Patient education	Counselling on the value of sticking to an exercise schedule. Instruction was given about reducing the fall risk in specific people.
Improve muscle strength and associate functional limitations	PNF exercises (D1-D2 flexion-extension pattern) and static, eccentric muscle contractions coupled with progressively increased resistance were employed. Rhythmic initiation patterns followed by a combination of isotonic followed by stabilizing reversal patterns were used (15 reps×3 sets); graded resistance exercise training.
Improve functions of facial muscles	Facial PNF exercises, facial massage, and facial exercises commenced (20 reps×2 sets).
Improve resting tremors	Dual-task training; functional electrical stimulator for 30 minutes; resistance training exercises (20 reps×2 sets); eccentric limb exercises (33-45 mins/session).
Increase aerobic capacity	Static bicycle cycling (20 mins); regular walking on the grounds.
Improve dynamic balance	Multidimensional balance training; balance exercises on unstable support surfaces.
Improve walking gait	Treadmill training with partial body weight (to prevent falls) followed by obstacle walking activity on the treadmill (30-40 mins/day) ad virtual reality and exergame exercises (20 mins/day).

Follow-up and outcome measures

The patient was given treatment for six weeks. Pre- and post-rehabilitation reflex and tone examination are summarized in Table 2 and Table 3. Manual muscle testing (MMT) and range of motion (ROM) assessment findings are shown in Table 4. Outcome measures after rehabilitation are given in Table 5.

**Table 2:** Pre- and post-intervention reflex examination ++: normal; +++: exaggerated

Reflexes	Pre intervention		Post intervention	
	Right	Left	Right	Left
Biceps jerk	++	++	++	++
Triceps jerk	++	++	++	++
Knee jerk	+++	++	++	++
Ankle jerk	++	++	++	++
Plantar response	Extensor	Extensor	Flexor	Flexor

**Table 3:** Pre- and post-intervention tone examination 1+: decreased tone; 2+: normal tone; 3+: increased tone

Muscles	Pre intervention		Post intervention	
Shoulder	Right	Left	Right	Left
Flexors	2+	2+	2+	2+
Extensors	2+	2+	2+	2+
Abductors	2+	2+	2+	2+
Adductors	2+	2+	2+	2+
Elbow				
Flexors	2+	2+	2+	2+
Extensors	2+	2+	2+	2+
Wrist				
Flexors	2+	3+	2+	2+
Extensors	2+	1+	2+	2+
Ulnar deviators	2+	3+	2+	2+
Radial deviators	2+	1+	2+	2+
Fingers				
Flexors	2+	3+	2+	2+
Extensors	2+	1+	2+	2+

**Table 4:** Pre- and post-rehabilitation MMT and ROM MMT: manual muscle testing; ROM: range of motion MMT grading: Grade 0: no contraction; Grade 1: flickering of contraction; Grade 2: full ROM in gravity-eliminated movement; Grade 3: full ROM against gravity; Grade 4: full ROM against gravity with minimum resistance; Grade 5: full ROM against gravity with maximum resistance

Muscles	Pre rehabilitation	Post rehabilitation
Shoulder flexors	2/5	3/5
Shoulder extensors	2/5	4/5
Shoulder abductors	2/5	3/5
Elbow flexors	3/5	4/5
Elbow extensors	3/5	4/5
Wrist flexors	2/5	4/5
Hip flexors	3/5	4/5
Hip extensors	2/5	4/5
Hip abductors	3/5	4/5
Knee flexors	3/5	4/5
Knee extensors	3/5	4/5
Ankle plantar flexors	3/5	4/5
Ankle dorsiflexors	3/5	4/5
Joint movement	Pre rehabilitation	Post rehabilitation
Shoulder flexion	0-70º	0-130º
Shoulder abduction	0-70º	0-130º
Elbow flexion	0-110º	0-130º
Wrist flexion	0-45º	0-60º
Hip flexion	0-80º	0-100º
Hip abduction	0-30º	0-40º
Knee flexion	0-70º	0-100º
Ankle plantarflexion	0-30º	0-40º
Ankle dorsiflexion	0-20º	0-30º

**Table 5:** Both prior to and following intervention outcomes

Outcome measures	Pre intervention	Post intervention
Unified Parkinson's Disease Rating Scale	100/260	200/260
World Health Organization Quality-of-Life Scale	50/100	80/100
Barthel Index	40/100	70/100

## Discussion

The substantia nigra, also known as the "trusted source region," is the region where nerve cells, or neurons, can be injured or die, causing the disorder. This area of the brain is essential for regulating movement. Dopaminergic neurons are found in the substantia nigra. This implies that they are in charge of dopamine production. A person may start to develop movement-related issues, such as tremors, rigidity, slowness of movement, and poor balance, all signs of PD, if they are unable to synthesize dopamine. Bradykinesia, stiffness, resting tremors, and postural instability are the four fundamental and significant clinical symptoms. This study's objective was to show how PD patients can benefit from PT rehabilitation. Tomlinson et al. conducted a study that explained that despite pharmacological treatments and surgical procedures, people who have PD continue to face increasing disability. The role of physiotherapy is to maximize functional ability and minimize secondary complications through movement rehabilitation within a context of education and support for the whole person. The overall aim is to optimize independence, safety, and well-being, thereby enhancing quality of life [5]. Radder et al. conducted a meta-analysis in 2020, which gives a thorough summary of the data supporting the efficacy of various physiotherapy therapies in PD treatment, enabling patients and physicians to choose a course of treatment based on the best available data [3]. A study by Okada et al. offered proof that patients with PD benefit from long-term physical treatment in terms of their motor symptoms and anti-Parkinsonian medication dosage, which may encourage the use of long-term PT [11]. A study by Benatru et al. showed that Parkinson's illness frequently affects postural stability. Postural irregularities are a result of axial motor involvement. Apart from late-onset idiopathic PD and atypical Parkinsonian syndromes, postural dysfunction often results in clinical deterioration in the disease's most severe stages [9]. A systemic review by Shahien et al. stated that patients with PD can improve their hand tremors using an assortment of PT methods. Numerous PT techniques, including electrical incitement, weightlifting, exercise, virtual reality, and transcranial low-voltage pulsed electromagnetic pulses, demonstrated encouraging hand tremor reduction results [12]. A systemic review by Barry et al. showed that exergames had been established in preliminary research as a feasible strategy for enhancing motor symptoms in individuals with PD; however, data on its safety and clinical efficacy are currently sparse [13].

A meta-analysis by Goodwin et al. found that physical functionality, strength, balance, the pace of walking, and quality of life in terms of health are all improved in PD patients [14]. A systemic review by Rocha et al. showed that there is potential for PD rehabilitation with exercise gaming treatment [15]. A study by Maranesi et al. concluded that with its integration of cognitive and physical activities inside a virtual, enhanced, or interactive gaming environment, exergaming-based technological rehabilitation might be more innovative than the traditional approach [16]. A systemic review by Garcia-Agundez et al. concluded that exergame-based therapy has been shown to be at least as safe, feasible, and successful as conventional PD rehabilitation [17]. A study by Cikajlo and Peterlin Potisk concluded that immersion in three-dimensional (3D) technology may lead to higher interest and enjoyment levels, which would improve functional performance more quickly and effectively [18]. A randomized controlled trial by Ribas et al. concluded that for PD, exergaming improved balance and decreased weariness [19]. A systemic review by Lei et al. stated that while multicenter randomized controlled research with larger sample sizes and more thoroughly designed, regularized interventions are required to give a stronger evidence-based basis to verify the possible advantages of virtual reality, virtual reality is probably a more effective form of rehabilitation for people with PD [20].

## Conclusions

Based on the earlier reviewed study, we inferred that physical treatment, which works on gaining functional recovery and improving quality of life, is crucial for achieving optimal functional recovery in patients with PD. Based on a case report, it has been concluded that integrative neurophysiotherapy can be effective in improving the symptoms, functional recovery, and quality of life of patients with PD. The rehabilitation included physiotherapy approaches like proprioceptive neuromuscular facilitation for limbs and face, dual-task training, and an electrical stimulator for improving tremors. Further, approaches like static cycling and regular walking on the ground were incorporated to improve aerobic capacities. Additionally, multidimensional training was included to improve dynamic trunk balance, followed by gait training. At the end of our rehabilitation, our patient showed improvement in the outcome measures used (the Unified Parkinson's Disease Rating Scale, the Barthel Index, and the World Health Organization Quality-of-Life Scale). Furthermore, the overview of systemic reviews suggests that exergames prove beneficial in addressing balance and gait impairments as well as enhancing life satisfaction in individuals with PD. It also highlights the importance of exergame exercises for improving symptoms in PD patients.
